# The Impact of Parental Depressive, Anxiety, and Stress Symptoms on Adolescents’ Mental Health and Quality of Life: The Moderating Role of Parental Rejection

**DOI:** 10.3390/children11111361

**Published:** 2024-11-09

**Authors:** Eirini Sofrona, Georgios Giannakopoulos

**Affiliations:** Department of Child Psychiatry, School of Medicine, National and Kapodistrian University of Athens, Aghia Sophia Children’s Hospital, 11527 Athens, Greece; ggiannak@med.uoa.gr

**Keywords:** children and adolescents, parental rejection, parental depressive, anxiety, stress symptoms, quality of life, mental health problems

## Abstract

**Background/Objectives**: Parental internalizing issues, particularly maternal mental health, can significantly influence adolescents’ mental health by altering parenting behaviors and roles. This study explores the role of parental rejection in moderating the relationship between parental depressive, anxiety, and stress symptoms and adolescents’ mental health and quality of life. **Methods**: One hundred thirty eight mothers (mean age: 47.96, SD = 5.06 years) and 68 fathers (mean age: 51.07, SD = 5.53 years) of adolescents aged 12–18 completed measures on mental health, parental rejection, and adolescent well-being. **Results**: Maternal rejection was found to moderate the relationship between maternal anxiety symptoms and adolescents’ quality of life, as well as between maternal anxiety symptoms and adolescents’ mental health problems. Paternal rejection moderated the relationship between paternal stress symptoms and adolescents’ mental health. However, parental rejection did not moderate the relationship between parental depression and adolescents’ quality of life or mental health. **Conclusions**: These findings highlight the distinct roles of maternal and paternal rejection in adolescent development and suggest that parental mental health, particularly anxiety and stress, has a significant impact on adolescent well-being. Future studies should consider the influence of both parents and focus on longitudinal effects. These results emphasize the need for interventions addressing parental rejection to improve adolescent outcomes.

## 1. Introduction

It is well established that parental behavior has a profound impact on a child’s psychosocial development, functioning either as a protective factor or as a risk factor for the development of psychosocial difficulties [1]. Parental practices characterized by increased discouragement, rejection, over-control, overprotection, or neglect are strong predictors of anxiety and depressive symptoms in developing children [2,3,4,5]. Rohner [6] identified two distinct dimensions of parental behavior: acceptance and rejection. These behaviors lie on a continuum ranging from warmth, affection, and support to the absence or significant lack of these qualities, coupled with behaviors that may harm the child physically or psychologically. Parental acceptance is linked to better psychosocial adjustment, whereas parental rejection is associated with mental health challenges [7,8,9,10]. While evidence suggests a strong association between parental rejection and adolescent mental health, the direction of this relationship remains unclear. It is possible that the relationship is bidirectional, with parental rejection exacerbating adolescent mental health problems and adolescents with mental health difficulties perceiving their parents as more rejecting [11,12]. The cross-sectional design of most studies, including this one, limits the ability to establish causality in this relationship.

Parental psychopathology, particularly internalizing symptoms such as anxiety, depression, and stress, can disrupt parenting practices and negatively affect parent–child interactions, leading to internalizing and externalizing problems in children [13,14,15]. Parents with high levels of anxiety, stress, or depression are more likely to exhibit rejecting behaviors towards their children [16,17,18,19]. Experiencing such rejection during adolescence—a critical developmental stage marked by significant cognitive, emotional, and relational changes—may lead adolescents to perceive their environment as hostile or threatening. This undermines their self-esteem, fosters feelings of helplessness, and contributes to the development of negative self-perceptions, anxiety, and depressive symptoms [20].

Several studies have supported the link between parental depression or anxiety and adolescent mental health symptoms, with maternal rejection having a partial mediating effect on this relationship, while paternal rejection did not show the same impact [21,22,23]. Similarly, Papp et al. [24] found that the degree of parental acceptance or rejection partly explains the association between parental stress and psychological distress in adolescents. On the other hand, Johnco et al. [25] identified parental rejection as an independent factor associated with anxiety and depressive symptoms in childhood and adolescence, but it did not significantly explain the relationship between parental internalizing problems and children’s symptoms.

These studies highlight a strong connection between parental mental health, parenting practices, and the mental health and development of children. However, research on this topic is marked by significant heterogeneity in conceptual definitions. For instance, “parental anxiety” and “depression” are sometimes used to refer to subclinical symptoms, while on other occasions, they refer to clinically diagnosed disorders [26]. Furthermore, studies use varying definitions of parenting behaviors, with some referring to general “parenting,” while others discuss specific behaviors such as warmth, control, and overprotection. Parental rejection is only occasionally identified as a distinct parenting behavior, and its definition and assessment tools differ across studies.

Another limitation in the literature is the frequent use of broad terms like “parental psychopathology” or “parental depression and anxiety”, without distinguishing between maternal and paternal influences [27]. While the role of maternal behavior in child development is well established [26], research on the influence of paternal behavior on adolescents’ quality of life and mental health has produced conflicting results, with some studies finding only weak effects [28,29].

Additionally, the number of studies examining the impact of parental mental health issues and negative parenting behaviors on adolescents’ quality of life is limited, and no study has explicitly included parental stress as a variable of interest.

In this context, the present study aims to further investigate maternal and paternal rejection and their role in the relationships between parental depressive, anxiety, and stress symptoms and adolescents’ quality of life and mental health. We conceptualized parental rejection as a possible moderator rather than a mediator in the relationship between parental mental health and adolescent outcomes. While past studies have often treated parental rejection as a mediator—acting as the mechanism through which parental mental health affects adolescent outcomes—our focus was on how parental rejection may interact with parental depressive, anxiety, and stress symptoms to influence adolescent mental health and quality of life, based on previous evidence mentioning that parental rejection acts more as an independent risk factor for adolescent mental health issues rather than a mediator [25]. This moderating role allows us to examine whether parental rejection amplifies or buffers the effects of parental mental health on adolescents. Acknowledging both moderating and mediating pathways in future research could provide a more comprehensive understanding of these relationships.

## 2. Materials and Methods

### 2.1. Participants and Procedures

This was a cross-sectional, non-interventional study conducted among parents with at least one adolescent child aged 12–18 years. The inclusion criteria required that the adolescent be between 12 and 18 years old and that the parents have a sufficient understanding of the Greek language to complete the questionnaires. A total of 206 parents (138 mothers and 68 fathers) of adolescents aged 12–18 from the general population participated in the study. Only Greek-speaking parents were included in this study to ensure the use of psychometrically validated versions of the assessment tools. The tools used are specifically validated for Greek-speaking populations, which ensures reliable and accurate data collection. Expanding the sample to non-Greek speakers would have required further translation and validation efforts, which were beyond the scope of this study. Regarding the sample size, to achieve a statistical power of 0.80, which is recommended for detecting medium effect sizes (Cohen’s f^2^ = 0.15), an estimated sample size of approximately 85–90 participants per group would be required, based on G*Power (version 3.1.9.6) calculations for multiple regression analyses with three predictors. With a total sample size of 206 participants, the study had sufficient power to detect medium to large effects but may have been underpowered to detect smaller effect sizes, particularly in subgroups.

Data collection took place through online self-administered questionnaires. The questionnaire was disseminated primarily through targeted social media platforms, including Facebook groups and other online communities specifically aimed at parents of adolescents (e.g., “Parents”, “Parents and child”, “Secondary education”). Additionally, the link was distributed via email and messaging platforms (i.e., Viber, WhatsApp) to further ensure the participation of parents. While some snowball sampling occurred, as participants were encouraged to share the survey within their networks, the majority of the dissemination focused on parenting-related social media groups to ensure the sample consisted of parents with adolescent children. A survey link was shared, and informed consent was obtained from all participants before they completed the questionnaires. A detailed description of the study’s objectives, the voluntary nature of participation, and assurances of confidentiality and anonymity were provided on the first page of the online survey. Participants were required to give their consent by clicking an agreement button before proceeding with the questionnaire. No responses were recorded until informed consent was provided.

The data collection period spanned from November 2023 to March 2024, and the entire process was conducted electronically using Google Forms. The study adhered to ethical standards and ensured confidentiality and voluntary participation at all stages of the data collection.

### 2.2. Measures

#### 2.2.1. Sociodemographic Data

Participants provided information on their gender, age, and the gender and age of their adolescent child. Additionally, data were collected on marital status, place of residence, educational level, socioeconomic status, and the adolescent’s school grade and country of birth.

#### 2.2.2. Parental Depression, Anxiety, and Stress

Parental depressive, anxiety, and stress symptoms were assessed using the Greek version of the Depression Anxiety Stress Scale (DASS-21) [30,31]. This self-report scale comprises twenty-one items, divided into three subscales with seven items each, evaluating depression, anxiety, and stress. Responses are given on a 4-point Likert scale ranging from 0 (does not apply to me at all) to 3 (applies to me most of the time). Each subscale score is calculated by summing the item responses and multiplying the total by 2. Higher scores indicate greater severity of symptoms. The internal consistency of the DASS-21 in this study was high, with α = 0.90 for depression, α = 0.85 for anxiety, and α = 0.89 for stress, indicating good reliability.

#### 2.2.3. Parental Rejection

The Parental Acceptance/Rejection Questionnaire (PARQ—Short Form—Parent Version) [32] was used to evaluate parental acceptance/rejection. This questionnaire contains 24 items rated on a 4-point Likert scale, with responses ranging from 1 (never) to 4 (always). The PARQ assesses four dimensions of parental behavior: (a) warmth/affection (8 items, reverse-scored to indicate coldness/lack of affection), (b) hostility/aggression (6 items), (c) indifference/neglect (6 items), and (d) undifferentiated rejection (4 items). The scores are summed to generate a total rejection score, with higher scores indicating greater parental rejection. The internal consistency of the scale was high (α = 0.87).

#### 2.2.4. Adolescent Quality of Life

Adolescents’ quality of life was assessed using the Greek version of the KIDSCREEN-27 (Parent Version) [33]. This questionnaire consists of 27 items that evaluate five dimensions of the child’s life over the past two weeks: (a) physical well-being (5 items), (b) psychological well-being (7 items), (c) autonomy and parental relationships (7 items), (d) peer relationships and social support (4 items), and (e) school environment (4 items). Responses are given on a 5-point Likert scale ranging from 1 (never) to 5 (always). The total score provides a general index of health-related quality of life, with higher scores indicating a better quality of life. The internal consistency of the KIDSCREEN-27 was excellent (α = 0.91).

#### 2.2.5. Adolescent Mental Health Problems

Adolescent mental health issues were evaluated using the Greek version of the Strengths and Difficulties Questionnaire (SDQ) [34,35]. The SDQ is a screening tool for the early detection of mental health problems in children and adolescents. In this study, the parent-reported version was used to assess the adolescent’s emotional and behavioral difficulties over the past six months. The SDQ consists of 25 items, grouped into five subscales: emotional symptoms, conduct problems, hyperactivity/inattention, peer relationship problems, and prosocial behavior. Each item is rated on a 3-point Likert scale (0 = not true and 2 = certainly true). Subscale scores for emotional symptoms, conduct problems, hyperactivity/inattention, and peer relationship problems are summed to create a total difficulty score, where higher scores indicate greater difficulties. As per the SDQ guidelines, the prosocial behavior subscale is not included in the total difficulty score but is analyzed separately, with higher scores indicating more positive behavior. The internal consistency of the total difficulties score in the present study was acceptable (α = 0.69).

### 2.3. Statistical Analysis

Data were analyzed using the Statistical Package for the Social Sciences (SPSS) version 29.0. Initially, the normality of the distributions for all study variables was assessed using the Kolmogorov–Smirnov test. Descriptive statistics, including means and standard deviations, were calculated for all variables under study. The internal consistency of the scales was evaluated using Cronbach’s alpha coefficient. Statistical significance was set at *p* < 0.05 for all analyses.

To examine potential differences in parental depressive, anxiety, and stress symptoms, parental rejection, adolescent quality of life, and adolescent mental health problems based on demographic variables, independent-samples *t*-tests and Mann–Whitney U tests were used for two-group comparisons. One-way ANOVA and Kruskal–Wallis tests were applied for comparisons involving more than two groups.

Moderation analyses were conducted to test whether parental rejection moderated the relationship between parental depressive, anxiety, and stress symptoms and adolescents’ quality of life and mental health problems. The moderation effects were tested using the PROCESS macro (version 4.0) for SPSS [36]. This tool allows for the simultaneous testing of multiple moderating and/or mediating variables, accommodating both simple and complex models. In this analysis, the following were examined: (a) whether the independent variable (parental depressive, anxiety or stress symptoms) predicted the dependent variable (adolescents’ quality of life or mental health), (b) whether the proposed moderator (parental rejection) predicted the dependent variable, and (c) whether the interaction between the independent variable and the moderator predicted the dependent variable. A significant interaction effect would indicate that parental rejection serves as a moderator in the relationship between parental depressive, anxiety or stress symptoms and adolescents’ quality of life or mental health. In the present study, 5000 bootstraps were employed for the estimation of confidence intervals. Bootstrapping is a non-parametric resampling technique, which does not require the assumption of normality. This method generates robust confidence intervals based on the resampled data, making it suitable for analyzing data regardless of their distribution.

## 3. Results

### 3.1. Sociodemographic Characteristics

The sample consisted of 206 parents, including 138 mothers and 68 fathers, with an average age of 47.96 years (SD = 5.06) for mothers and 51.07 years (SD = 5.53) for fathers. The adolescents, for whom the parents reported data, had an average age of 15.47 years (SD = 1.96), with 51.9% of them being boys and 48.1% girls. Most of the parents were married (90.3%), with the remaining being divorced or widowed. Most participants lived in urban areas (76.2%), with a smaller portion residing in suburban (14.6%) or rural areas (9.2%). In terms of socioeconomic status, 64.1% of the families identified as being of average financial means, and 26.2% considered themselves above average. Detailed sociodemographic characteristics are presented in Table 1.

### 3.2. Data Normality Assessment

To assess data normality, we conducted the Kolmogorov–Smirnov (KS) test, which is known to be sensitive to larger sample sizes. As expected, the KS test returned significant values for several variables. To further evaluate normality, we also examined skewness and kurtosis values, which were within the acceptable range (±2) for most variables, indicating approximate normality. Given the minor deviations from normality, we employed bootstrapping in the moderation analysis (via the PROCESS macro), which did not rely on normality assumptions.

### 3.3. Descriptive Statistics and Psychosocial Variables

The mean depression score for the sample was 6.09 (SD = 7.61), for anxiety, it was 4.31 (SD = 6.51), and for stress, it was 9.61 (SD = 8.03). Similarly, the mean parental rejection score for the sample was 32.49 (SD = 8.29), while for adolescents’ perceived quality of life, the mean score was 104.64 (SD = 12.23), and for the adolescents’ mental health problems, it was 8.08 (SD = 5.22).

Comparisons between mothers and fathers showed that mothers reported significantly higher levels of depressive (U = 3898, *p* = 0.045), anxiety (U = 3722.5, *p* = 0.012), and stress (U = 3847, *p* = 0.035) symptoms compared to fathers. Additionally, mothers reported a better adolescent quality of life (t_(204)_ = 2.043, *p* = 0.042) compared to fathers. No significant differences were found between mothers and fathers in their reports of parental rejection or adolescent mental health problems.

### 3.4. Differences in Psychosocial Outcomes by Demographic Variables

Analyses examining the influence of demographic factors revealed that the gender of the adolescent was significantly associated with overall mental health difficulties, with parents reporting higher levels of difficulties for boys (U = 4399, *p* = 0.035). Younger adolescents (aged 12–15 years) were reported to have more mental health difficulties compared to older adolescents (aged 16–18 years) (U = 4277.5, *p* = 0.018), and younger adolescents also had a lower reported quality of life (t_(204)_ = −2.121, *p* = 0.035). Parents with lower educational levels reported higher anxiety (U = 1969, *p* = 0.036), while no significant differences were found for family status, place of residence, or socioeconomic status on the psychosocial variables.

### 3.5. Moderation Analyses

#### 3.5.1. Parental Depressive Symptoms

The results indicated that parental depressive symptoms and rejection, as well as their interaction, explained 20.4% of the variance in adolescents’ quality of life (R^2^ = 0.204, F_(3, 201)_ = 17.21, *p* < 0.001). For mothers, depressive symptoms and rejection together explained 27% of the variance in adolescents’ quality of life (R^2^ = 0.27, F_(3, 133)_ = 16.41, *p* < 0.001), while for fathers, the explained variance was lower at 7.8% (R^2^ = 0.078, F_(3, 64)_ = 1.81, *p* = 0.155). Parental rejection did not significantly moderate the relationship between parental depressive symptoms and adolescent quality of life (Table 2).

Similarly, parental depressive symptoms and rejection explained 32.4% of the variance in adolescent mental health problems (R^2^ = 0.324, F_(3, 201)_ = 32.17, *p* < 0.001). Maternal depressive symptoms and rejection explained 42.6% of the variance in adolescent mental health issues (R^2^ = 0.426, F_(3, 133)_ = 32.90, *p* < 0.001), while paternal depressive symptoms and rejection explained 18% of the variance (R^2^ = 0.18, F_(3, 64)_ = 4.67, *p* = 0.005). Parental rejection did not serve as a significant moderator in the relationship between parental depressive symptoms and adolescent mental health (Table 3).

#### 3.5.2. Parental Anxiety Symptoms

Parental anxiety symptoms and rejection, along with their interaction, explained 18.6% of the variance in adolescents’ quality of life (R^2^ = 0.186, F_(3, 201)_ = 15.31, *p* < 0.001). Maternal anxiety symptoms and rejection together explained 27.5% of the variance (R^2^ = 0.275, F_(3, 133)_ = 16.80, *p* < 0.001), while paternal anxiety symptoms and rejection explained 8.7% (R^2^ = 0.087, F_(3, 64)_ = 2.04, *p* = 0.118). Notably, maternal rejection significantly moderated the relationship between maternal anxiety symptoms and adolescents’ quality of life (β = 0.03, *p* = 0.027), with the interaction explaining an additional 3% of the variance (ΔR^2^ = 0.03, F_(1, 133)_ = 4.98, *p* = 0.027) (Table 4).

Parental anxiety symptoms and rejection also explained 34% of the variance in adolescent mental health problems (R^2^ = 0.34, F_(3, 201)_ = 34.52, *p* < 0.001). Maternal anxiety symptoms and rejection explained 45% of the variance (R^2^ = 0.45, F_(3, 133)_ = 36.24, *p* < 0.001), and paternal anxiety symptoms and rejection explained 24.2% (R^2^ = 0.242, F_(3, 64)_ = 6.83, *p* < 0.001). Parental rejection significantly moderated the relationship between parental anxiety symptoms and adolescent mental health (β = −0.01, *p* = 0.047), with maternal rejection explaining an additional 6% of the variance in mental health problems (ΔR^2^ = 0.06, F_(1, 133)_ = 13.44, *p* < 0.001) (Table 5).

#### 3.5.3. Parental Stress Symptoms

Parental stress symptoms and rejection explained 19.1% of the variance in adolescents’ quality of life (R^2^ = 0.191, F_(3, 201)_ = 15.78, *p* < 0.001). For mothers, stress symptoms and rejection explained 26.3% of the variance (R^2^ = 0.263, F_(3, 133)_ = 15.83, *p* < 0.001), while for fathers, stress symptoms explained 7.6% (R^2^ = 0.076, F_(3, 64)_ = 1.77, *p* = 0.164). Parental rejection did not significantly moderate the relationship between parental stress symptoms and adolescent quality of life (Table 6).

However, parental stress symptoms and rejection explained 30.2% of the variance in adolescent mental health problems (R^2^ = 0.302, F_(3, 201)_ = 28.98, *p* < 0.001). Maternal stress symptoms and rejection together explained 39.9% of the variance (R^2^ = 0.399, F_(3, 133)_ = 29.46, *p* < 0.001), while paternal stress symptoms and rejection explained 23.6% (R^2^ = 0.236, F_(3, 64)_ = 2.84, *p* = 0.006). Notably, paternal rejection significantly moderated the relationship between paternal stress symptoms and adolescent mental health problems (β = 0.04, *p* = 0.006), with the interaction explaining an additional 10% of the variance (ΔR^2^ = 0.10, F_(1, 64)_ = 8.06, *p* = 0.006) (Table 7). 

## 4. Discussion

This study aimed to explore the role of parental rejection—both maternal and paternal—in moderating the relationships between parental depressive, anxiety, and stress symptoms and adolescents’ quality of life and mental health problems. The findings highlight the significance of parental rejection, particularly maternal rejection, in influencing these relationships, with important implications for understanding the impact of parental mental health on adolescent outcomes.

First, parental rejection was found to be a significant predictor of both adolescent quality of life and mental health problems, for both mothers and fathers. This confirms previous findings that parental rejection can have a lasting negative impact on adolescent well-being [10]. However, the degree of influence varied between maternal and paternal rejection. While maternal rejection had a stronger moderating effect on the relationship between parental anxiety symptoms and adolescents’ mental health, paternal rejection showed a more limited role, particularly in moderating the relationship between paternal stress symptoms and adolescents’ mental health problems. Previous studies support the greater influence of the mother on the caregiving and psychosocial development of the offsprings, emphasizing her role as the primary caregiver who exerts a stronger influence on the child’s development through her behavior [5,15,28]. However, there are also some studies that highlight the equally significant impact of both parents on adolescents’ mental health [37,38]. In Greek families, mothers traditionally play a more central role in caregiving and emotional involvement, and studies have shown that maternal behaviors often have a stronger impact on children’s psychological development compared to paternal behaviors [39,40]. This may explain why maternal rejection was found to exert a greater influence on adolescent mental health outcomes in our sample. However, as paternal roles evolve in modern Greek society, future research may reveal more balanced effects between maternal and paternal influences.

The results showed that parental depressive symptoms significantly predicted adolescent quality of life and mental health issues. Maternal depressive symptoms were a strong predictor, while paternal depressive symptoms did not significantly affect either quality of life or mental health outcomes in adolescents. This suggests that maternal depressive symptoms may play a more critical role in shaping adolescent mental health, a finding that aligns with previous research emphasizing the maternal role in child development [5,15]. Interestingly, parental rejection did not moderate the relationship between parental depressive symptoms and adolescent outcomes. This finding is consistent with the study of Johnco et al. [25], who also did not find a moderating effect of parental rejection on the depression–adolescent outcome relationship, though they did not differentiate between maternal and paternal influences.

With respect to parental anxiety symptoms, no direct predictive relationship was found between anxiety symptoms and adolescent quality of life. However, maternal rejection moderated the relationship between maternal anxiety symptoms and adolescent quality of life, indicating that maternal anxiety symptoms may have an indirect effect on adolescents when rejection is also present. These findings highlight the role of maternal rejection as a critical factor in the interaction between maternal anxiety symptoms and adolescent outcomes. Similarly, parental anxiety symptoms significantly predicted adolescent mental health problems, with maternal rejection again serving as a significant moderator. These results are consistent with Ma and colleagues, who found that maternal rejection mediated the relationship between maternal anxiety and adolescent anxiety but did not find a similar effect for paternal rejection [21].

Parental stress symptoms did not significantly predict adolescents’ quality of life, and parental rejection did not moderate this relationship. This finding was consistent for both mothers and fathers, suggesting that parental stress symptoms alone may not directly influence adolescents’ perceived quality of life. However, parental stress symptoms significantly predicted adolescent mental health problems, particularly for mothers. Notably, paternal rejection moderated the relationship between paternal stress symptoms and adolescent mental health issues despite the lack of a direct effect of paternal stress symptoms. This suggests that paternal rejection may exacerbate the impact of stress on adolescent mental health, even if the direct influence of paternal stress is limited. These findings are consistent with existing research that points to the complex role of paternal behavior in adolescent development [28,29]. Furthermore, the findings related to parental stress symptoms revealed significant associations with adolescent mental health problems but not with quality of life. This may be due to the different pathways through which stress affects parenting behaviors. While stress may manifest in ways that increase conflict or reduce emotional availability, impacting mental health directly, its influence on broader aspects of adolescents’ quality of life may be more diffuse. Moreover, the moderation effect of parental rejection, particularly paternal rejection, amplifies the impact of parental stress symptoms on adolescent mental health outcomes, highlighting the importance of parental behaviors in this relationship. These findings are consistent with previous research, which shows that stress can exacerbate negative parenting behaviors, leading to adverse outcomes in children [18,19]. Future research should further explore these dynamics to fully understand how parental stress affects different dimensions of adolescent well-being.

Although this study highlights the significant role of parental rejection, particularly maternal rejection, in moderating the relationship between parental anxiety and stress symptoms and adolescent outcomes, the cross-sectional nature of the study limits the ability to explore these impacts over time and establish causal relationships. It also remains unclear whether parental rejection leads to increased mental health problems in adolescents or whether adolescents with existing mental health issues perceive their parents as more rejecting. This bidirectional relationship has been suggested by previous research [11,12]. A longitudinal study design would be more appropriate for providing clearer insights into the direction of these relationships and examining the long-term impact of parental mental health and rejection on adolescent mental health and quality of life.

The heterogeneity in the literature regarding the role of parental rejection as a moderator in the relationship between parental psychopathology and adolescent mental health is reflected in these findings. Some studies, such as Reigstad et al.’s [22], have found that maternal rejection plays a significant mediating role in this relationship, whereas others, like Kim’s [23], did not observe such an effect. The differences in findings across studies may be due to variations in study design, sample characteristics, and measurement tools. For example, the current study focused on adolescent mental health, while other studies have examined younger children or focused on different aspects of mental health, such as externalizing or internalizing problems.

The absence of significant effects of parental rejection in moderating the relationship between parental depressive symptoms and adolescent outcomes could be attributed to the sample size or the relatively lower frequency of depressive symptoms reported in this non-clinical population. Anxiety and stress symptoms were more prevalent among the parents in this study, which might explain why parental rejection played a more significant moderating role in these relationships. Additionally, depressive symptoms tend to be less frequently reported in the general population compared to anxiety and stress, as seen in this study, which may further limit the ability to detect significant moderating effects of parental rejection.

Furthermore, it is possible that parental rejection, when examined in isolation, may not be sufficient to account for the complex dynamics of parental psychopathology and adolescent outcomes. A combination of multiple negative parenting behaviors—such as overcontrol, strictness, and harsh punishment—may have a stronger moderating or even mediating effect on the relationship between parental depressive, anxiety, and stress symptoms and adolescent mental health and quality of life. Future studies should consider examining these additional dimensions of parenting behavior to provide a more comprehensive understanding of their role.

Finally, the differential effects observed between maternal and paternal rejection in moderating the relationship between parental stress symptoms and adolescent mental health suggest that gender-specific dynamics may be at play. This finding aligns with previous research, which has shown that maternal behavior often has a stronger impact on adolescent mental health compared to paternal behavior, particularly in cultures where mothers are traditionally the primary caregivers, suggesting that the differential influence of maternal and paternal rejection observed in our study may reflect broader cultural and familial dynamics [5,15,28,39,40]. The smaller number of fathers in this study may have limited the statistical power to detect significant effects. 

This study has several limitations that should be noted. First, one major limitation of this study is its cross-sectional design, which prevents causal conclusions and does not allow for the determination of the direction of the relationship between parental rejection and adolescent mental health. It is likely that this relationship is reciprocal. Furthermore, the cross-sectional design prevents the exploration of long-term effects of parental mental health and rejection on adolescent outcomes. A longitudinal design would be better suited for investigating these relationships over time, exploring the causal pathways between parental behaviors and adolescent mental health outcomes and understanding the developmental trajectories of adolescent mental health in response to parental behaviors. Future research should aim to use longitudinal methodologies to provide a more comprehensive understanding of these processes.

Second, the exclusive use of self-report scales in the present study introduces the possibility of social desirability bias and errors in reporting. Although the questionnaires were completed anonymously to reduce these biases, and we used well-validated tools with high reliability, the reliance on self-reports may still affect the accuracy of the data. Additionally, the fact that this study relies solely on parental reports, which may not fully capture the adolescents’ experiences and perceptions of their own mental health and quality of life, adds to the limitations of this study. Collecting data directly from adolescents was not part of the current study design. Future research could address these limitations by adopting a multi-method approach, incorporating data from multiple sources such as adolescent self-reports, teacher or clinician observations, or objective measures where feasible. This would provide a more comprehensive and less biased view of adolescent mental health and quality of life, while offering deeper insights into how adolescents perceive their mental health and the impact of parental behaviors.

Another limitation of this study is the gender imbalance in the sample, with a higher number of mothers (138) compared to fathers (68), which may limit the generalizability of the results, particularly in terms of paternal influences. This discrepancy is commonly observed in parenting research, as mothers are more likely to participate in studies involving family and child outcomes. Although a more balanced sample would provide further insights, increasing the number of fathers in this study was not feasible due to recruitment difficulties. Future research should aim to recruit more fathers to better understand paternal influences on adolescent mental health and quality of life. Additionally, the study focused on a non-clinical, Greek-speaking population, which may not fully represent other cultural or clinical contexts. Moreover, the study’s use of the DASS-21 to assess subclinical symptoms rather than diagnosed mental health conditions further limits the findings. The measurement of parental stress was not detailed enough to capture its complexity. To capture the complexity of parental stress, future studies could employ more detailed instruments, such as the Parental Stress Scale (PSS) or the Parenting Stress Index (PSI). These tools assess specific domains of stress, including financial, work-related, and parenting-related stressors, providing a more nuanced understanding of how different types of stress influence parental behaviors and adolescent outcomes.

Finally, future research could benefit from analyzing adolescent mental health outcomes based on the individual subscales of the Strengths and Difficulties Questionnaire (SDQ), such as emotional symptoms, conduct problems, hyperactivity/inattention, and peer relationship problems. This would allow for a more detailed exploration of the specific domains of adolescent mental health that are most influenced by parental behaviors, providing more targeted insights into how parental rejection and other factors impact different aspects of adolescent well-being.

## 5. Conclusions

This study emphasizes the role of parental rejection, particularly maternal rejection, in the relationship between parental anxiety symptoms and adolescent outcomes. The findings suggest that while maternal rejection plays a significant moderating role, paternal rejection also influences adolescent mental health, albeit to a lesser extent. These results highlight the need for further research into the specific mechanisms through which maternal and paternal behaviors affect adolescent development. Additionally, interventions aimed at reducing parental rejection, particularly in families where parental anxiety or stress is high, could be beneficial in improving adolescent mental health and quality of life.

## Figures and Tables

**Table 1:** Sociodemographic characteristics of parents and adolescents.

		Total Sample		Mothers		Fathers	
		*n*	%	*n*	%	*n*	%
Marital status	Married	186	90.3	124	89.9	62	91.2
	Other	20	9.7	14	10.1	6	8.8
Place of residence	Urban	157	76.2	103	74.6	54	79.4
	Suburban	30	14.6	22	16.0	8	11.8
	Rural	19	9.2	13	9.4	6	8.8
Adolescents’ country of birth	Greece	205	99.5	137	99.3	68	100.0
	Other	1	0.5	1	0.7	0	0.0
Adolescents’ level of education	Primary school	18	8.7	12	8.7	6	8.8
	Junior high school	79	38.3	52	37.7	27	39.7
	High school	109	53.0	74	53.6	35	51.5
Parents’ level of education	Primary education	1	0.5	0	0.0	1	1.5
	Secondary education	28	13.6	18	13.0	10	14.7
	Tertiary education	177	85.9	120	87.0	57	83.8
Socioeconomic status	High	54	26.2	32	23.2	10	32.4
	Middle	132	64.1	96	69.6	36	52.9
	Low	20	9.7	10	7.2	22	14.7

**Table 2:** Regression coefficients for the relationship between parental depressive symptoms and adolescents’ quality of life, moderated by parental rejection.

					95% CI	
		*Β*	*t*	*p*	Lower	Upper
Total sample	Depressive symptoms	−0.24	−2.19	0.030	−0.46	−0.02
	Rejection	−0.55	−5.61	0.000	−0.74	−0.36
	Depressive symptoms × rejection	−0.01	−0.68	0.497	−0.03	0.015
Mothers	Depressive symptoms	−0.27	−2.14	0.034	−0.52	−0.02
	Rejection	−0.62	−5.31	0.000	−0.85	−0.39
	Depressive symptoms × rejection	0.001	0.05	0.963	−0.03	0.03
Fathers	Depressive symptoms	−0.10	−0.45	0.654	−0.57	0.36
	Rejection	−0.42	−2.23	0.030	−0.80	−0.04
	Depressive symptoms × rejection	−0.003	−0.07	0.941	−0.08	0.07

**Table 3:** Regression coefficients for the relationship between parental depressive symptoms and adolescents’ mental health problems, moderated by parental rejection.

					95% CI	
		*Β*	*t*	*p*	Lower	Upper
Total sample	Depressive symptoms	0.20	4.52	0.000	0.11	0.28
	Rejection	0.27	7.10	0.000	0.20	0.35
	Depressive symptoms × rejection	−0.002	−0.37	0.713	−0.01	0.01
Mothers	Depressive symptoms	0.24	4.85	0.000	0.14	0.33
	Rejection	0.31	6.87	0.000	0.22	0.40
	Depressive symptoms × rejection	−0.01	−1.76	0.081	−0.02	0.001
Fathers	Depressive symptoms	0.16	1.72	0.091	−0.03	0.34
	Rejection	0.25	3.25	0.002	0.10	0.40
	Depressive symptoms × rejection	0.02	1.38	0.173	−0.01	0.05

**Table 4:** Regression coefficients for the relationship between parental anxiety symptoms and adolescents’ quality of life, moderated by parental rejection.

					95% CI	
		*Β*	*t*	*p*	Lower	Upper
Total sample	Anxiety symptoms	−0.15	−1.12	0.262	−0.41	0.11
	Rejection	−0.64	−6.21	0.000	−0.85	−0.44
	Anxiety symptoms × rejection	0.02	1.17	0.245	−0.01	0.04
Mothers	Anxiety symptoms	−0.24	−1.70	0.091	−0.52	0.04
	Rejection	−0.78	−6.37	0.000	−1.02	−0.54
	Anxiety symptoms × rejection	0.03	2.23	0.027	0.004	0.06
Fathers	Anxiety symptoms	0.24	0.83	0.408	−0.34	0.82
	Rejection	−0.45	−2.39	0.020	−0.82	−0.07
	Anxiety symptoms × rejection	0.01	0.29	0.773	−0.07	0.10

**Table 5:** Regression coefficients for the relationship between parental anxiety symptoms and adolescents’ mental health problems, moderated by parental rejection.

					95% CI	
		*Β*	*t*	*p*	Lower	Upper
Total sample	Anxiety symptoms	0.26	5.16	0.000	0.16	0.36
	Rejection	0.30	7.43	0.000	0.22	0.37
	Anxiety symptoms × rejection	−0.01	−1.99	0.047	−0.02	0.00
Mothers	Anxiety symptoms	0.27	5.08	0.000	0.17	0.38
	Rejection	0.37	7.98	0.000	0.28	0.46
	Anxiety symptoms × rejection	−0.02	−3.67	0.000	−0.03	−0.01
Fathers	Anxiety symptoms	0.26	2.34	0.023	0.04	0.49
	Rejection	0.21	2.89	0.005	0.06	0.35
	Anxiety symptoms × rejection	0.03	1.83	0.073	−0.003	0.06

**Table 6:** Regression coefficients for the relationship between parental stress symptoms and adolescents’ quality of life, moderated by parental rejection.

					95% CI	
		*Β*	*t*	*p*	Lower	Upper
Total sample	Stress symptoms	−0.18	−1.73	0.086	−0.39	0.03
	Rejection	−0.57	−5.72	0.000	−0.77	−0.38
	Stress symptoms × rejection	0.001	0.06	0.950	−0.02	0.02
Mothers	Stress symptoms	−0.21	−1.71	0.089	−0.45	0.03
	Rejection	−0.70	−5.52	0.000	−0.95	−0.45
	Stress symptoms × rejection	0.02	1.29	0.200	−0.01	0.04
Fathers	Stress symptoms	−0.05	−0.23	0.816	−0.45	0.36
	Rejection	−0.43	−2.01	0.049	−0.86	−0.002
	Stress symptoms × rejection	−0.002	−0.07	0.945	−0.07	0.07

**Table 7:** Regression coefficients for the relationship between parental stress symptoms and adolescents’ mental health problems, moderated by parental rejection.

					95% CI	
		*Β*	*t*	*p*	Low	High
Total sample	Stress symptoms	0.15	0.04	0.001	0.06	0.23
	Rejection	0.27	0.04	0.000	0.19	0.35
	Stress symptoms × rejection	0.002	0.004	0.616	−0.006	0.01
Mothers	Stress symptoms	0.19	4.01	0.000	0.10	0.29
	Rejection	0.31	6.31	0.000	0.22	0.41
	Stress symptoms × rejection	−0.01	−1.49	0.139	−0.02	0.003
Fathers	Stress symptoms	0.07	0.90	0.371	−0.09	0.23
	Rejection	0.35	4.19	0.000	0.18	0.51
	Stress symptoms × rejection	0.04	2.84	0.006	0.01	0.06

## Data Availability

The data presented in this study are available on request from the corresponding author due to privacy and legal reasons.
